# The foodscape: classification and field validation of secondary data sources across urban/rural and socio-economic classifications in England

**DOI:** 10.1186/1479-5868-9-37

**Published:** 2012-04-02

**Authors:** Amelia A Lake, Thomas Burgoine, Elaine Stamp, Rachael Grieve

**Affiliations:** 1 Centre for Public Policy and Health, School of Medicine and Health, Wolfson Research Institute Durham University Queen's Campus, Thornaby, Stockton-on-Tees TS17 6BH, UK; 2 UKCRC Centre for Diet and Activity Research (CEDAR), Box 296, University of Cambridge, Institute of Public Health, Forvie Site, Robinson Way, Cambridge CB2 0SR, UK; 3 Institute of Health & Society, The Baddiley-Clark Building, Newcastle University, Newcastle NE2 4HH, UK; 4 Human Nutrition Research Centre, Institute of Health & Society, Newcastle University, Newcastle NE2 4HH, UK

**Keywords:** Foodscape, Food environment, Secondary data, Urban, Rural, Socio-economic status, Ground-truthing, Validation

## Abstract

**Background:**

In recent years, alongside the exponential increase in the prevalence of overweight and obesity, there has been a change in the food environment (foodscape). This research focuses on methods used to measure and classify the foodscape. This paper describes the foodscape across urban/rural and socio-economic divides. It examines the validity of a database of food outlets obtained from Local Authority sources (secondary level & desk based), across urban/rural and socio-economic divides by conducting fieldwork (ground-truthing). Additionally this paper tests the efficacy of using a desk based classification system to describe food outlets, compared with ground-truthing.

**Methods:**

Six geographically defined study areas were purposively selected within North East England consisting of two Lower Super Output Areas (LSOAs; a small administrative geography) each. Lists of food outlets were obtained from relevant Local Authorities (secondary level & desk based) and fieldwork (ground-truthing) was conducted. Food outlets were classified using an existing tool. Positive predictive values (PPVs) and sensitivity analysis was conducted to explore validation of secondary data sources. Agreement between 'desk' and 'field' based classifications of food outlets were assessed.

**Results:**

There were 438 food outlets within all study areas; the urban low socio-economic status (SES) area had the highest number of total outlets (n = 210) and the rural high SES area had the least (n = 19). Differences in the types of outlets across areas were observed. Comparing the Local Authority list to fieldwork across the geographical areas resulted in a range of PPV values obtained; with the highest in urban low SES areas (87%) and the lowest in Rural mixed SES (79%). While sensitivity ranged from 95% in the rural mixed SES area to 60% in the rural low SES area. There were no significant associations between field/desk percentage agreements across any of the divides.

**Conclusion:**

Despite the relatively small number of areas, this work furthers our understanding of the validity of using secondary data sources to identify and classify the foodscape in a variety of geographical settings. While classification of the foodscape using secondary Local Authority food outlet data with information obtained from the internet, is not without its difficulties, desk based classification would be an acceptable alternative to fieldwork, although it should be used with caution.

## Background

Despite being a relatively new field of research [1], interest in the influence of the food environment on eating behaviours and its relationship with obesity has increased. Food environments are seen to be an important driver of obesity and measurement issues are of high importance [2]. Food and beverages consumed outside of the home are associated with higher energy intakes than foods prepared at home and are of importance across all age groups [3]. Dietary behaviours are an important contributing factor to socioeconomic inequalities in overweight/obesity [4]. Studies and reviews of the literature have reported issues around measurement complexities [5,6] and the need to have 'reliable and valid measures' of the food environment [7]. However, in this field, there is little information about issues of validity and measurement error [2,8].

Due to the differences in international contexts (both cultural and physical) [9], this paper will mainly focus on UK studies and UK evidence. Two recent UK papers have validated secondary sources of food environment data, i.e. lists of food outlets, from Local Authority and commercial sources within an urban setting [10,11]. However, both highlighted the need to repeat the validation in different geographic contexts. Recently, in the US, the first validation of rural areas has been published [8]. This paper aims to meet this need by exploring the field validation (also known as 'ground-truthing' or 'on the ground verification' [12]) of secondary data from Local Authority sources (Environmental Protection Records) in urban and rural areas as well as within areas of high and low socio-economic status, in the UK.

In the UK, few studies have addressed the topic of how food retailing influences diet [13]. While the concept of food deserts are not thought to apply to the UK context [14], it is recognised that in the UK there are differences in access and availability to food according to socio-economic standing [15-17]. For example, Macdonald et al. [17] described a 'concentration' effect whereby fast food chains were concentrated in more deprived areas of England and Scotland. However the difference in food access and food availability between urban and rural settings in the UK has received less attention. In Scotland, Smith et al. [18], used four environmental settings (island, rural, small town and urban) to explore access to grocery stores by environment. In contrast to much US research, Smith et al. [18] found that the residents of the most deprived areas had shorter travel times to grocery stores compared with those living in the least deprived areas. Smith et al. [18]concluded that the relationship between deprivation and accessibility to grocery stores at a neighbourhood level varies by environmental setting, and therefore requires researchers and policy makers to be context specific when dealing with issues of neighbourhood exposure to diet. Despite this studies in rural regions are rare. In the US, Sharkey and Horel [19] found the most common type of food outlet in the 6-county rural region of Texas (11,567 km^2^) to be convenience stores, also reported earlier by Liese et al. [20]. Again in the US, Powell et al. [21] reported that rural areas had the least number of food outlets of all types, but especially a shortage of chain supermarkets, compared to other areas. They reported that urban areas had over seven times as many supermarkets compared to rural areas. In New Zealand, Pearce et al. [22] reported that urban low SES areas had good access to multinational and local fast food outlets however low SES rural areas had the least access.

Fieldwork (primary data collection/direct method) to verify and record a particular food environment or 'foodscape' is recognised as the gold standard measure [23]. However, this fieldwork process is both time and labour intensive and is thus not practical for large areas. The alternative is the use of secondary data, which can be obtained from a range of sources. However obtaining, cleaning and preparation of this secondary data is not without its issues [24]. For example using Yellow Pages data, described by Burgoine et al. [25] and Lake et al. [11], required manual data input, address and postcode checking and food outlet re-classification. Cummins and Macintyre [10], Macdonald et al. [26] and Lake et al. [11] used Environmental Protection records held by Local Authorities. The latter study [11] also developed a detailed classification tool to describe the wide range of food environments available in the UK; the classification tool has 22 main categories with 78 more detailed subsections. The aim was to develop a food outlet classification tool that could be used to classify the food environment from both direct field observations and secondary data sources. The development of this classification system and a review of existing systems has been described by Lake et al. [11].

When researchers obtain secondary data on the food environment, particularly for larger geographical areas, where a number of organisations provide data and may all be using different systems to classify their food outlets, there is often a need to reclassify the food outlets in a uniform way [8,11]. Classification of food outlet type is considerably easier using visual observation techniques, compared with trying to classify an outlet based on its name alone or using data obtained from the internet. While there is confidence in the reliability of secondary data sources [10-12,27], no previous UK study has described how easily or reliably a desk based researcher can classify and describe the food environment for a UK geographical area by use of secondary sources alone and not verifying the 'type' of food outlet during a field visit.

This paper aims to compare the foodscape across urban and rural areas as well as within areas of high and low socio-economic status. Secondly, it will explore the field validation of secondary data from Local Authority sources (Environmental Protection Records) in urban and rural areas in the North East of England as well as within areas of high and low socio-economic status using sensitivity and positive predictive values (PPV). The third aim of this study was to test the efficacy of desk based classification using a pre-defined 22 point food outlet classification tool [11] compared with ground-truthing. This third aim will examine if the categorisation of outlets based on data obtained from the name and internet searching are substantiated in reality.

## Methods

### Study areas

Six study areas were purposively selected: high/low socio-economic status (SES), irrespective of urbanity/rurality (two areas) and urban/rural, high/low SES (four areas). These study areas covered a range of area types, as described, but were few enough (six) for study within the time frame for this research. The definition of a 'study area' was based upon the boundaries of the Lower Super Output area (LSOAs), a small administrative geography of which there are 32,482 in England. LSOAs are homogeneous in containing roughly 1,500 individuals and are small enough to represent spatial patterns in a nuanced manner, however they are not necessarily equal in terms of their size. As the foodscapes of the study areas were to be systematically audited 'on foot' by the researcher, and research time was constrained, smaller, similar sized areas were favoured for the research, as were areas that were accessible to the researcher in terms of proximity (Newcastle/Sunderland and vicinity); this said, the selection of study areas was biased towards those with a high number of food outlets to yield more accurate results and to maximise the potential differences found between areas.

**Figure 1:**
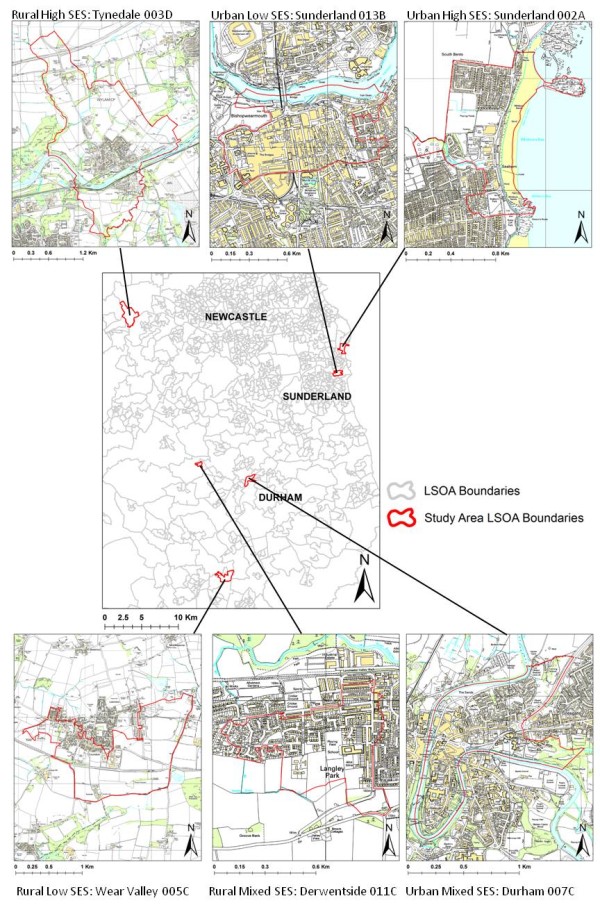
**Locations of LSOA study areas (n = 6) throughout North East England**. ^© ^Crown Copyright/database right 2011. An Ordnance Survey/EDINA supplied service.

The urban/rural classification was based on Department for Communities and Local Government recommendations, which define small towns, villages and hamlets with less than 10,000 residents as 'rural' [28], with data obtained from National Statistics. SES was assessed using the Index of Multiple Deprivation (IMD) 2007 [29]. The IMD is a compound measure of socio-economic status, combining aspects of employment, health, crime, living environment, education, housing and income, at the LSOA level. IMD scores (that increase as deprivation increases) for England were ranked from most deprived to least and quartiled, as to create comparable groups of LSOAs, with low/high SES study areas drawn from the most/least deprived quartiles, respectively. The study areas were (1) urban mixed SES, LSOA code Durham 007C; (2) rural mixed SES, Derwentside 011C; (3) urban high SES, Sunderland 002A; (4) urban low SES, Sunderland 013B; (5) rural high SES, Tynedale 003D; (6) rural low SES, Wear Valley 005C (Figure 1).

### Data

Data on the locations of food outlets were collected as part of an ongoing Economic and Social Research Council (ESRC) studentship, between February and August 2009 [24]. Data were provided, upon Freedom of Information (FOI) request from Environmental Health departments in Local Authorities in the North East of England, between February and August 2009. The locations of all outlets retailing food were specifically requested. In order to facilitate routine hygiene and food safety inspections, such records are collected and maintained by Environmental Health Departments. As all food vendors are required to register their premises with their Local Authority by law, this dataset is assumed to be the most accurate source of 'foodscape' data available [11]. This data obtained from the Local Authorities is referred to as the 'secondary data'. One researcher (thus negating any inter-rater bias in data collection) was trained in the use of the outlet classification tool [11], data collection and analysis techniques. The outlets were classified as per a 22-point classification system developed by Lake et al.[11] (Table 1). This system was previously developed as a culturally relevant and detailed system for classifying the food environment [11]. In order to classify food outlets, the type of outlet and the foods available therein needed to be known [11]. Classifications 'at the desk' were made using internet searches (October - November 2009). If the outlet name alone provided insufficient detail to allow classification, other available information (such as postcode) was used within search engines such as Google and directories such as Yell.com, in order to provide further insight. Following the desk based classification, the six study areas were systematically audited (fieldwork) by the trained researcher for the locations of food outlets present in the field. This involved a systematic method of surveying the areas by foot and by vehicle. Food outlets were noted as being present/absent from the Local Authority data, and any food outlets present in new locations were also noted. 'Field based' classifications of store type were made using the aforementioned classification tool. This fieldwork was conducted October - November 2009, meaning that there was relatively little time lag between Local Authority data acquisition (secondary data) and field validation.

**Table 1:** 22 point Classification tool for food outlets used in the investigation (see Lake et al. [11])

Classification	
1	Restaurant

2	Pub/Bar

3	Convenience

4	Supermarket

5	Takeaway Food

6	Work Place/Education

7	Hotels/Function Rooms/Associations

8	Medical e.g. Pharmacy

9	Entertainment e.g. cinema, bowling, theatre, sports venues

10	Department Stores i.e. large retail store organised into departments offering variety of merchandise.

11	Discount Stores

12	Fast Food

13	Pizzeria

14	Non-Food Stores/Novelty Items e.g. clothes/accessory shops, gift shops, stationery shops, cosmetic/toiletry shops.

15	Food Production Services e.g. wholesalers, suppliers, distributers, caterers, cash & carry

16	Sandwich Shop

17	Café/Coffee Shop

18	Specialist e.g. organic food stores, holistic food stores, fair trade stores, oriental food stores

19	Specialist Traditional e.g. Delicatessen, Butcher, Baker, Fishmonger, Confectioners, Greengrocer

20	Baker-Retail Freshly baked savouries/bread, pre-made sandwiches, baked sweet products & branded products. Usually a chain, takeaway only.

21	Health and Leisure e.g. Gyms, Health Clubs, Leisure Centre

22	Other

### Statistical analysis

Agreement between 'desk' and 'field' based classifications of food outlets were assessed using a binomial test (non-parametric) in SPSS version 17. Positive predictive values (PPVs) were calculated to assess the proportion of the Local Authority data that was also present in the field; sensitivity analysis was used to assess the proportion of the food outlets existing in reality that were accounted for in the Local Authority data (see Lake et al.[11] for further detail of PPV & sensitivity analysis). Fisher's exact test (used because of small expected values) was used to detect differences in percentage agreement - between Local Authority data and field data - across socio-economic and urban/rural divides.

## Results

### Comparison of the foodscape according to geographic areas

A total of 438 outlets selling food were recorded. The number and percentage of total outlets (Local Authority food outlets plus outlets observed in the fieldwork) examined in each study area are shown in Table 2. The urban low SES area had the highest number of total outlets (n = 210, 47.9%) with the rural high SES area having the least (n = 19, 4.3%).

**Table 2:** Number and percentage of total food outlets (Local Authority list plus field work) in each study area

Area	Number	Percent
**Urban mixed SES**	134	30.6

**Rural mixed SES**	25	5.7

**Rural low SES**	22	5.0

**Rural high SES**	19	4.3

**Urban low SES**	210	47.9

**Urban high SES**	28	6.4

**Total**	438	100.0

The urban/rural classification was based on Department for Communities and Local Government recommendations [28]. SES was assessed using the Index of Multiple Deprivation (IMD) 2007 [29].

The areas were approximately the same geographic size, however the population sizes of the areas ranged from 1401 in the urban high SES area to 5024 in the urban mixed SES area. In the urban low SES area, food outlets were clustered very tightly together, with each street having numerous food outlets. There was very little residential housing in this area. In the rural high SES area, the opposite was found. Food outlets were dispersed throughout the area and the majority of streets and estates were residential.

### Number of outlet types using classification tools

The highest numbers of outlet types using the classification tool at the desk and in the field are shown in Table 3. Outlets that were not present (either in the Local Authority list or in the field) were excluded when determining the highest frequencies in each study area. There were some similarities and differences between the desk and field based classifications of the outlets in the rural mixed SES, rural high SES and urban low SES areas.

**Table 3:** Highest number of outlet type (and as a percentage of all outlets) using desk and field based classification tools in each area

Study area	Outlet classification - desk based	Outlet classification - field based
**Urban mixed SES**	Restaurant (n = 25; 23%)	Restaurant (n = 27; 20%)

**Rural mixed SES**	Convenience (n = 4; 20%)	Convenience (n = 4; 16%); Takeaway (n = 4; 16%)

**Rural low SES**	Takeaway (n = 4; 20%)	Pub/Bar (n = 4; 29%)

**Rural high SES**	Hotels/Function rooms/Associations (n = 4; 25%)	Pub/Bar (n = 4; 25%); Hotels/Function rooms/Associations (n = 4; 25%)

**Urban low SES**	Pub/Bar (n = 28; 15%); Café/ Coffee shop (n = 28; 15%)	Pub/Bar (n = 43; 23%)

**Urban high SES**	Restaurant (n = 7; 30%)	Restaurant (n = 8; 32%)

However, the highest number of outlets in the rural low SES area were different using the desk and field based tools; takeaways using the desk based method (20% of all outlets), but the fieldwork indicated Pubs/Bars to be the most frequent classification (29%). The fieldwork indicated the most frequent outlets found in the urban high SES area to be Restaurants (32%), while in the rural high SES the most frequent classification was Pub/Bar and Hotels/Function rooms (each 25%). In both the urban and rural low SES areas Pub/Bar was the most frequent classification type (23% and 29% respectively).

### Validation of secondary sources

#### Presence of the food outlets: local authority data compared with fieldwork

**Figure 2:**
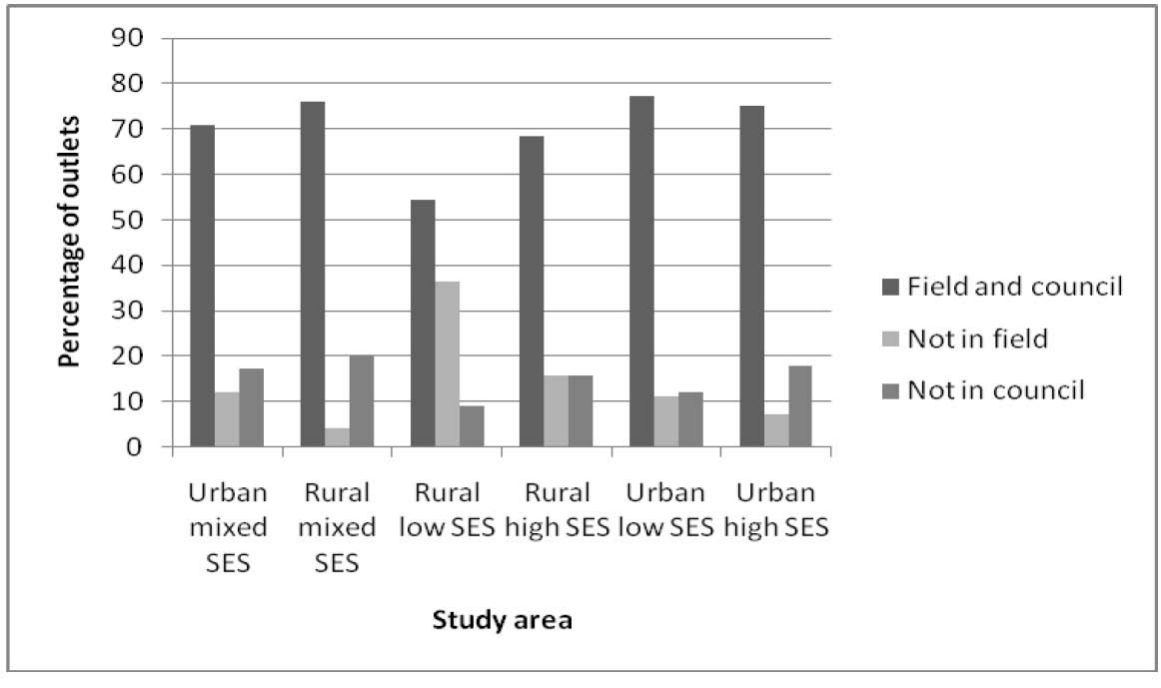
**Presence of outlets in the Local Authority list and the fieldwork**.

Figure 2 illustrates the presence of the food outlets in the Local Authority list compared with what was present in the field. In the rural low SES area, 36.4% of the total outlets recorded in the Local Authority list were not present in the field. There was a high number of outlets across all areas that were not present in the Local Authority list but which were present the field. These outlets were only identified through ground-truthing.

#### Positive predictive value analysis: local authority data compared with fieldwork

The positive predictive value (PPV) was used to calculate the accuracy of the Local Authority's food outlet data in representing the actual food environment present in reality using fieldwork as a gold standard. An ideal PPV of 100% would mean that all the outlets found in the field were also listed in the Local Authority food outlet data. Table 4 demonstrates that there was a range of PPV values obtained, with the highest PPV for urban low SES areas (87%) and the lowest for Rural mixed SES (79%).

**Table 4:** Positive Predictive Values (PPV) and Sensitivity for each study area plus sensitivity categories adapted from Paquet et al. [12]

Study Area	PPV (%)	Sensitivity (%)	Sensitivity categories	Sensitivity range (%)
**Urban mixed SES**	81	86 [Good]	**Very poor**	< 20

**Rural mixed SES**	79	95 [Excellent]	**Poor**	21-30

**Rural low SES**	86	60 [Moderate]	**Fair**	31-50

**Rural high SES**	81	81 [Good]	**Moderate**	51-70

**Urban low SES**	87	88 [Good]	**Good**	71-90

**Urban high SES**	81	91 [Excellent]	**Excellent**	> 90

#### Sensitivity analysis: local authority data compared with fieldwork

Using the fieldwork as the gold standard, sensitivity was assessed. Sensitivity categories were taken from Paquet et al.[12]. Sensitivity between the outlets listed in the Local Authority data that were also in the field was highest in the rural mixed SES area (95%). However in the rural low SES area, sensitivity was moderate (60%). Table 4 illustrates that both the rural mixed SES and urban high SES areas had excellent sensitivity.

#### Validation of desk based classification versus field classification

Each food outlet in the Local Authority food outlet list was classified (i.e. placed within one of 22 classification groups) with the aid of the internet. However, in both the rural and urban high and low SES areas, some outlets could not be classified as there was no information available about them (n = 13). The percentages of food outlets classified in the Local Authority food outlet list using each of the data sources in each study area is shown in Table 5. For example, in the urban mixed SES area, 32.8% of the Local Authority food outlets were classified using Yell.com (a commercial search directory) with only 3.7% classified using their own websites. In all of the study areas apart from the urban mixed SES area, the majority of the food outlets in the Local Authority list could be classified using the name of the individual outlet alone (for example, WH Smith or McDonalds). Yell.com was used to classify the majority of outlets in the Local Authority list in the urban mixed SES area and was also used frequently to classify food outlets in the Local Authority list in the other areas when the name alone was not sufficient. In the urban mixed SES area and the urban high SES area the highest number of outlets classified using the classification tool were the same at the desk and in the field (Table 3).

**Table 5:** Percentages of food outlets classified in the Local Authority food outlet list using each of the additional internet data sources in each study area

	Additional data sources						
**Study Area**	*Could not classify	**Name only**	**Yell commercial directory**	**Thomson local commercial** **directory**	**Google**	**Own website**	**Not in Local Authority data**

**Urban mixed SES**	0	18.7	32.8	7.5	20.1	3.7	17.2

**Rural mixed SES**	0	56.0	12.0	0	12.0	0	20.0

**Rural low SES**	13.6	36.4	13.6	4.5	22.7	0	9.1

**Rural high SES**	5.3	31.6	26.3	10.5	10.5	0	15.8

**Urban low SES**	2.9	43.3	26.2	4.3	11.0	0.5	11.9

**Urban high SES**	10.7	35.7	32.1	0	3.6	0	17.9

*even with the use of the internet we were unable to classify this outlet from the desk alone.

#### Testing the accuracy of desk based classification compared to field based classification (agreement assessment)

The accuracy of the initial desk based classification of the foodscape with the aid of internet sources was analysed by comparing the *agreement *between the desk based classification of the food outlets and the fieldwork classification of the food outlets. Outlets that were excluded from this analysis included those not present in the field or those not listed in the Local Authority food outlet data. Outlets that could not be classified at the desk (deemed 'Unclassifiable') were included in this comparison so as not to bias the results. Percentage agreement is show in Table 6.

**Table 6:** Observed proportion of outlets in agreement or disagreement with the given classification using the 22-point classification tool at the desk compared with the field

			22-point classification tool	
**Study area**	**Number of outlets**	**Agreement**	**Observed proportion**	**p-value**

Urban mixed SES	134	Agree	0.89	< 0.001
			
		Disagree	0.11	

Rural mixed SES	25	Agree	0.89	0.001
			
		Disagree	0.11	

Rural low SES	22	Agree	0.83	0.039
			
		Disagree	0.17	

Rural high SES	19	Agree	0.85	0.022
			
		Disagree	0.15	

Urban low SES	210	Agree	0.78	< 0.001
			
		Disagree	0.22	

Urban high SES	28	Agree	0.80	0.012
			
		Disagree	0.20	

All areas	438	Agree	0.83	< 0.001
			
		Disagree	0.17	

All urban areas	372	Agree	0.82	< 0.001
			
		Disagree	0.18	

All rural areas	66	Agree	0.86	< 0.001
			
		Disagree	0.14	

All high SES areas	47	Agree	0.82	< 0.001
			
		Disagree	0.18	

All low SES areas	232	Agree	0.79	< 0.001
			
		Disagree	0.21	

In all the study areas, most outlets classified at the desk using the classification tool agreed with the field based classification. The highest percentage of agreement was in the rural mixed SES area (68%) and the lowest was in the rural low SES area (45.5%). However, there was not a great variation between urban and rural areas overall (Table 7). There were a fairly high percentage of outlets that could not be classified and compared in all areas, however importantly, all areas had few outlets that did not agree (< 16.3%). A Fishers exact test was used to test the significance of the agreement/disagreement between desk and field based classifications. The outlets that could not be *compared *because they were absent from the Local Authority list or absent in the field (the 'Neither' column in Table 7) were excluded in this statistical comparison. For example, an outlet classified as category 2 at the desk that was also classified as category 2 in the field was in agreement. However, if it was classified as category 5 in the field there was a disagreement in the given classifications. The observed proportions of outlets in agreement or disagreement within each study area were shown in Table 6.

**Table 7:** Percentage of outlets that agree, disagree or neither (not in field or/not in Local Authority data) using the 22 point classification tool at a desk and in the field

Study Area	Agree	Disagree	Neither in field or/LA data
**Urban mixed SES**	63.4	7.5	29.1

**Rural mixed SES**	68.0	8.0	24

**Rural low SES**	45.5	9.1	45.5

**Rural high SES**	57.9	10.5	31.6

**Urban low SES**	60.5	16.2	23.3

**Urban high SES**	57.1	14.3	28.6

**All areas**	60.5	12.6	26.9

**All urban**	61.0	13.2	25.8

**All rural**	57.6	9.1	33.3

**All high SES**	57.4	12.8	29.8

**All low SES**	58.6	15.9	25.4

There were significantly (*p *< 0.05) more outlets in agreement than disagreement in all study areas. For example, in the urban mixed SES area 89% of outlet classifications were in agreement and 11% were in disagreement (*p *< 0.05).

## Discussion

'Reliable' measures of the food environment have been described as the 'foundation' of research that will help to inform obesity related policy [1]. This research has highlighted that despite some difficulties, secondary data sources available in the UK provide an accurate picture of the urban/rural, high/low socio-economic status food environment, and that desk based classification is an acceptable alternative to fieldwork.

### Comparison of the foodscape according to geographic area

There is little information about the UK foodscape across urban/rural and SES divides. Despite the fact that all areas were approximately the same geographic size, the area that had the greatest number of food outlets was the urban low SES area (n = 210) and the area with the least number of outlets was the rural high SES (n = 19). The population sizes of the areas varied with a population of 1401 in the urban high SES area and 5024 in the urban mixed SES area. The observations and descriptive results during fieldwork highlighted the differences in the areas. There was clustering of outlets in the urban low SES area, and a lack of residential housing while the rural areas had outlets dispersed throughout. Likewise, Macdonald et al. [26], found that the least deprived areas of Glasgow were the least well served by food outlets and the most deprived were the best. However, despite the fact that these findings also hold true for high and low SES areas of North East England, Macdonald et al. [26] concluded that their least deprived areas did not necessarily have better access to food outlets and vice versa. In this study and in the work of Macdonald et al. [26] urban low SES areas having the greatest concentrations of food outlets does not necessarily mean that individuals in urban low SES areas had the best overall access to a healthy diet, or to wide variety of food types; future research will seek to examine these relationships further. In an earlier study also in Newcastle, White et al. [30] concluded 'there are inequalities in retail provision that are geographically patterned, but these are not necessarily all "bad"'. This statement holds true for other areas of North East England explored in the present study.

Cummins et al. [15] reported that the number of McDonald's fast food outlets was highest in the most deprived areas of England and Scotland compared to the least deprived. In the present study only three out of the 438 outlets investigated were classified as fast food outlets (not necessarily McDonalds but similar; one in the urban mixed SES area and two in the urban low SES area). There were no fast food outlets found in either of the high SES areas. In Glasgow, Cummins and Macintyre [31] reported that the most deprived areas had the most frozen food and discount food outlets. Similar results were found in the present study where the highest number of these outlet types was reported in the urban low SES area although none were found in the rural low SES area. In a recent review Beaulac et al. [5,14] concluded that 'there is little evidence that socioeconomically deprived areas of the UK are systematically disadvantaged by food deserts.' This study does not provide evidence that low SES areas are more at risk of food deserts than other areas as they have more food outlets compared to the higher SES areas.

Rural areas may be more disadvantaged compared to urban areas when it comes to food availability, as the rural areas have fewer food outlets that are dispersed. Availability or 'potential access' to food has both social and geographic elements. Sharkey [23] suggested that changes in the food environment such as price, variation and quality of food, and the size and numbers of food outlets are more likely to have negative effects on rural areas in parts of the U.S. In the present study, food outlets in rural areas (especially the mixed SES rural area) were more spread out, which may have an effect on where residents of rural areas purchase their food. As found elsewhere, this study also observed food outlets in urban areas to be more clustered. Smith et al. [18] found that people who live in the most deprived areas of Scotland had the shortest travelling distance to the nearest food store compared to the least deprived areas. The present study reported that the low SES areas had *more *outlets than the high SES counterparts. However the type of outlet differed between areas.

### Number of outlet types

The classification of the *types *of outlet differed between urban and rural areas and between SES of areas. In every study area the highest number of outlets could be defined as places where food is consumed away from the home such as takeaways, traditional restaurants or pubs/bars, rather than supermarkets or stores where foods can be purchased and prepared at home. This supports longitudinal work in Northumberland which also observed a high number of outlets of 'foods for consumption away from the home' [25]. The highest frequencies of outlets in all areas are probably more likely to sell higher calorie beverages and higher calorie and fat foods compared to what one might consume at home [3,25]. It is acknowledged that the relationship between an individual's food intake and the wider food environment is 'complex' [6,25] and merits further investigation.

### Validation of secondary sources: local authority list compared with fieldwork

The results of this study, covering a range of geographic areas, supports previous work in an inner city area by Lake et al. [11], which highlighted that there were a number of outlets in each study area that were neither present in the field or on the Local Authority data list. Those *not *on the Local Authority food outlet list could only be identified and classified in the fieldwork and vice versa. These findings suggest that in order to obtain the most accurate picture of the food environment, fieldwork must be conducted so that any changes or closures to outlets can be noted. However, it is likely that the food environment changes rapidly due to new food outlets opening or closing and so fieldwork would need to be carried out regularly if the food environment was to be studied over a longer period of time. In order to calculate how accurate the Local Authority's food outlet data actually was, sensitivity and PPV analyses were used. Although none of the areas had a PPV of 100%, all were fairly high (PPV > 78%), which suggested that the Local Authority food outlet data would be an acceptable alternative to fieldwork. The highest PPV was in the urban low SES area (PPV = 87%). These PPVs are slightly lower than the PPV of 91.5% obtained in inner city Newcastle by Lake et al. [11] using the same methods. In the current study both urban study areas had a PPV value of 81% and 87%. The sensitivities between the Local Authority food outlet data and the fieldwork were considered to be 'moderate' to 'excellent' in all study areas, which also suggested the Local Authority data is likely to be a satisfactory representation of the food environment. It is interesting to note the rural low SES area had a high PPV (86%), yet a 'moderate' sensitivity, which reflects the large number of missing outlets on the Local Authority list that were present in reality. It also serves to emphasise the importance of measuring both PPV and sensitivity. Given the extra time and resources required to conduct fieldwork (particularly in large rural areas), this paper argues that the use of Local Authority collected secondary data is a viable option, even in rural locales. This is in agreement with previous work [11], although secondary data sources (commercial as well as Local Authority) should always be used with caution and with appreciation for their potential limitations [8,10].

### Accuracy of desk based classification compared to fieldwork (agreement assessment)

In most large studies previously conducted in the UK, classification of the food environment was completed using secondary data sources alone [25,26]. However, very few studies have tested how reliable these data sources actually are using ground-truthing [11]. Additionally, in the UK, studies have not explored rural environments.

The classification tool used in this study was developed in the North East of England [11]. It is perhaps unsurprising that classification of food outlet types is more straightforward in the field than it is from the desk with only limited information. Although most of the outlets could be classified from their name (especially well known chains such as Tesco or WH Smith), some were more difficult (for example 'Lou Lous' food outlet, which was a 'traditional café/coffee shop') and required various additional data sources to make a classification. Some outlets were impossible to classify at the desk due to a lack of information. However, despite difficulties, in most areas there was agreement. In Glasgow, Cummins and Macintyre [2,10] reported that the level of agreement between secondary data and fieldwork observations was 'high but imperfect'. Similarly, in a US study [27], the level of agreement between a secondary and primary data collection method were found to be fairly high and significant. The researchers concluded that either method could be used with 'reasonable confidence' [27]. Interestingly, using the classification tool in the combined rural areas, significantly more outlets were in agreement than disagreement. This is surprising as many outlets in the rural areas could not be classified using any of the additional internet sources. Reasons for this are unclear, however it is possible that businesses in rural areas are small and may not feel the need to register with commercial directories or have a website of their own (as they only serve a limited number of local residents). Telephoning food outlets may assist in making accurate food outlet classifications; this approach will be used in future studies.

The agreement in each SES area across the urban and rural divides was similar whether it was the high or low SES. A different result from Cummins and Macintyre [10] who reported that most errors in agreement were made in the deprived neighbourhoods.

### Strengths and limitations

This study investigated the food environments of North East England, including urban and rural, as well as high and low SES area comparisons. Definitions of 'urban' and 'rural' were used according to Department for Communities and Local Government guidelines [28]. Whilst these definitions may not be transferable outside the UK, they are certainly an accurate measure of urbanity and rurality within the UK. An assessment of secondary food environment data across these divides in the UK has been absent in the literature to date, yet has been called for [10,11] and has only recently been published in the US [8].

The classification tool [11] was an appropriate way to measure the food environment and to categorise food outlets that were present. It contained enough detail to give each food outlet a very specific category based on the type and manner in which food was sold. A more detailed 78 point version of this tool exists. Use of this more detailed tool may have influenced the reliability of the classifications made both in the field and at the desk. In this study the inter-rater reliability of the tool was not tested, however all the data was collected by one trained researcher. In the urban mixed SES area of Durham city centre however, a major part of the food environment was not classified as it had previously been excluded on the grounds that it was a market, with stalls ordinarily registered to the owners' home addresses. However, this is a permanent indoor market that housed 12 food outlets of various kinds and observations suggested it to be a major source of food retail. Since the fieldwork was conducted, the classification tool has been further developed to include categories for such markets, ice cream and burger vans, in order to help produce a tool that can record the entirety of the food environment.

Whilst the LSOAs selected as study areas in this research were purposively sampled, in part for their geographic convenience, this was necessary as to ensure that the areas selected contained some food outlets within them to field validate. Many potential LSOAs in the surrounding area were devoid of food outlets altogether and were, for this reason, not a candidate for study. It may have been preferable to conduct a random sample of LSOAs for inclusion in the study, however this was not feasible on this occasion. This could be seen as a limitation. Furthermore, whilst it may have been preferable to 'ground truth' more than six LSOAs in total, this was also not possible. With this said, the LSOAs selected for study were as relatively highly populated with food outlets as was possible, allowing for detailed and substantive comparisons between study sites regardless.

Although this study has given an accurate and detailed picture of the food environments within these defined areas in terms of the category of outlets present, we did not examine the types of food and beverages sold within each outlet, nor individual purchasing patterns. Exploration of the price and nutritional profiles of products sold within each outlet in relation to the foodscape is currently being explored by this group and will give an even more detailed representation of the food environment.

Various difficulties arose when obtaining the field data. In the rural mixed SES and rural high SES areas the amount of land covered within the boundaries was too large to reasonably cover on foot, therefore a car had to be used. This was both labour and time intensive, and only serves to strengthen the call for an alternative and accurate source of food outlet data to be identified. In reality, it is difficult for researchers to ground-truth large geographic areas, thus secondary data sets are important resources.

## Conclusion

Classification of the food environment at a desk using secondary Local Authority food outlet data with the aid of additional internet searches is not without its difficulties. In most cases desk based classification would probably be an acceptable alternative to fieldwork although it should be used with caution. Fieldwork produces the most accurate and detailed representation of the food environment as a whole, but is time, cost and labour intensive. Differences in the foodscape between SES within the urban and rural divides were apparent however further work is needed to be done in order to determine if this is true for all areas of North East England (currently underway) and England and what effects such environments have on the health of the population exposed to them.

## Abbreviations

IMD: Index of multiple deprivation; LSOA: Lower super output areas; PPV: Positive predicative values

## Competing interests

The authors declare that they have no competing interests.

## Authors' contributions

AAL was principle investigator and researcher, and was responsible for devising the protocol, supervision of the data collection and analysis. AAL was lead author of the paper. RG collected the data and performed the data analysis. ES gave analysis advice and assisted with analysis. TB was responsible for all geographical elements of this study. Additionally TB co-supervised the data collection, analysis and write up. All authors critically reviewed the manuscript and approved the final version submitted for publication.

## Authors' information

The authors are from a range of disciplines. AAL and RG are nutritionists, TB a health geographer and ES a statistician
